# NLRP12 as a Regulator of Inflammation: Insights into the Correlation with Autoinflammatory Disorders

**DOI:** 10.3390/genes17040421

**Published:** 2026-04-01

**Authors:** Beatrice Rosa, Elisabetta Tabolacci, Roberta Pietrobono, Eugenio Sangiorgi, Fiorella Gurrieri, Pietro Chiurazzi, Ludovico Luca Sicignano, Elena Verrecchia, Maurizio Genuardi, Donato Rigante, Raffaele Manna

**Affiliations:** 1 UOC Genetica Medica, Fondazione Policlinico Universitario Agostino Gemelli IRCCS, 00168 Rome, Italy; beatrice.rosa03@icatt.it (B.R.); elisabetta.tabolacci@unicatt.it (E.T.); pietro.chiurazzi@unicatt.it (P.C.); maurizio.genuardi@unicatt.it (M.G.); 2 Department of Life Sciences and Public Health, Sezione di Medicina Genomica, Università Cattolica Sacro Cuore, 00168 Rome, Italy; roberta.pietrobono@unicatt.it; 3 UOSD Laboratorio di Genetica Medica, Ospedale Santa Rosa (Belcolle), 01100 Viterbo, Italy; eugenio.sangiorgi@asl.vt.it; 4 Research Unit of Medical Genetics, Department of Medicine, University Campus-Biomedico of Rome, 00128 Rome, Italy; f.gurrieri@unicampus.it; 5 Operative Research Unit of Medical Genetics, Fondazione Policlinico Universitario Campus Bio-Medico, 00128 Rome, Italy; 6 Department of Aging, Orthopaedical and Rheumatological Sciences, Fondazione Policlinico Universitario Agostino Gemelli IRCCS, 00168 Rome, Italy; ludovicoluca.sicignano@policlinicogemelli.it (L.L.S.); elena.verrecchia@policlinicogemelli.it (E.V.); 7 Periodic Fevers Research Center, Università Cattolica Sacro Cuore, 00168 Rome, Italy; donato.rigante@unicatt.it; 8 Department of Life Sciences and Public Health, Fondazione Policlinico Universitario Agostino Gemelli IRCCS, 00168 Rome, Italy

**Keywords:** NLRP12, autoinflammatory disorders, autoinflammation, recurrent fever, personalized medicine, innovative biotechnologies

## Abstract

**Background**: Dysregulation of the innate immune system is a key feature of autoinflammatory disorders, characterized by recurrent or chronic inflammation in the absence of high-titer autoantibodies and antigen-specific T cells. Among regulators of innate immunity, NLRP12 has emerged as an important modulator of inflammatory signaling pathways. As a member of the nucleotide-binding oligomerization domain-like receptor (NLR) family, NLRP12 negatively regulates nuclear factor (NF)-κB activity and contributes to immune homeostasis. However, the clinical significance of *NLRP12* variants and their association with disease phenotypes remain incompletely understood. This study aims to summarize current knowledge on the molecular role of NLRP12 and its involvement in autoinflammatory manifestations. **Methods**: A narrative review of the literature on NLRP12’s molecular functions and role in autoinflammatory diseases was performed. In addition, a cohort of 20 patients with recurrent fevers carrying *NLRP12* variants was analyzed from a clinical perspective, evaluating genetic findings and clinical features. **Results**: Available evidence indicates that NLRP12 regulates inflammatory signaling, particularly through modulation of NF-κB activity. Variants in the *NLRP12* gene have been associated with a spectrum of autoinflammatory phenotypes, ranging from periodic fever syndromes to broader systemic inflammatory manifestations. Clinical evaluation of the cohort confirmed the heterogeneity of disease presentations among individuals carrying *NLRP12* variants. **Conclusions**: NLRP12 plays an important role in the regulation of innate immune responses and may contribute to autoinflammatory phenotypes. Integrating molecular data with clinical observations may improve the understanding of *NLRP12* variants and support more accurate diagnostic and therapeutic strategies.

## 1. Introduction

Systemic autoinflammatory disorders (SAIDs) are heterogeneous diseases characterized by periodically recurrent inflammation, caused by variants in numerous genes that are involved in the control of innate immunity [1]. They share partially overlapping presentations characterized by recurrent fevers combined with variable inflammatory features, such as skin rashes, urticaria, arthromyalgia, headache, abdominal pain, and lymphadenopathy [1]. Age at onset may vary: symptoms often begin in early childhood—hence the importance for pediatricians to be familiar with these conditions—whereas they may also become evident at a later time [1,2]. It should be noted that accurately estimating the age at onset of these conditions is challenging, as precocious signs and symptoms are nonspecific, common, nuanced, hidden and confounding, leading to delay in the diagnostic suspicion. To date, different SAIDs have been described, such as familial Mediterranean fever (FMF; OMIM #249100), cryopyrin-associated periodic syndrome or NLRP3-associated autoinflammatory disease (OMIM #607115, #617772, #120100, #148200, #191900), mevalonate kinase deficiency (OMIM #260920), and tumor necrosis factor receptor-associated periodic syndrome (OMIM #142680), with all of them resulting from multiple pathological mechanisms involving pyrin- or NLRP3-inflammasomes [3,4,5,6,7].

**Figure 1:**
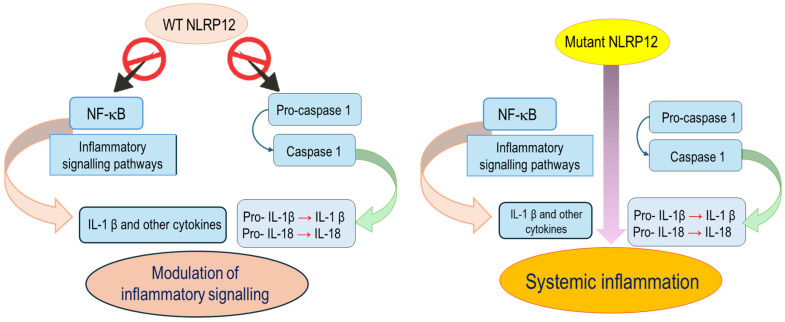
Overview of NLRP12-associated signaling pathways. Through inhibition of NF-κB signaling and modulation of caspase-1 activation, NLRP12 downgrades excessive inflammatory responses (**left**) and contributes to the pathogenesis of autoinflammatory manifestations when dysregulated (**right**).

In 2008, Jéru et al. reported the first two *NLRP12* variants in two unrelated families presenting a periodic fever [8]. The associated clinical features were consistent with the so-called NLRP12-associated autoinflammatory disease (NLRP12-AID), then redefined as “familial cold autoinflammatory syndrome 2” (or “FCAS2”; OMIM #611762), a rare autosomal dominant condition with variable penetrance, characterized by recurring fever, urticaria-like rashes, joint symptoms, headache and serologic evidence of inflammation during episodes, largely triggered by cold exposure. Abdominal and thoracic pain or sensorineural deafness have been less commonly described in this condition [9]. The *NLRP12* gene is located on chromosome 19 (19q13.42) and encodes for an intracellular NOD-like receptor (NLR); the full-length human *NLRP12* cDNA encodes for a 1062-amino acid protein, called *monarch-1*, with an estimated molecular weight of 120 kDa [10,11]. As with other members of the NLR family, the NLRP12 protein displays a tripartite structure comprising an N-terminal PYD that mediates homotypic protein–protein interactions for downstream signaling, a central NACHT (also known as NBD) domain, which plays a major role in ATP-dependent oligomerization, and a C-terminal leucine-rich repeat (LRR) domain, which acts in ligand sensing [10,12,13,14]. NLRP12 is mainly expressed in myeloid-lineage cells, including neutrophils, eosinophils, monocytes, macrophages, and immature dendritic cells, and its expression is reduced following exposure to different pathogens or their products [10,11,15,16]. NLRP12 is also known to act with a dual function in regulating innate immunity: depending on the context, it works as a negative regulator of inflammation *per se* or as a regulator of the NLRP3-inflammasome [12]. More specifically, NLRP12 negatively regulates NF-κB-dependent inflammatory signaling, thereby limiting inflammatory gene transcription and reducing downstream proinflammatory cytokine output. Otherwise, in some contexts, NLRP12 may modulate caspase-1 activation and therefore interleukin (IL)-1β/IL-18 production and release [4,12]. When NLRP12 is mutated, NK-κB-dependent signaling and caspase-1 cascade become hyperactivated [4,12] (Figure 1).

**Figure 2:**
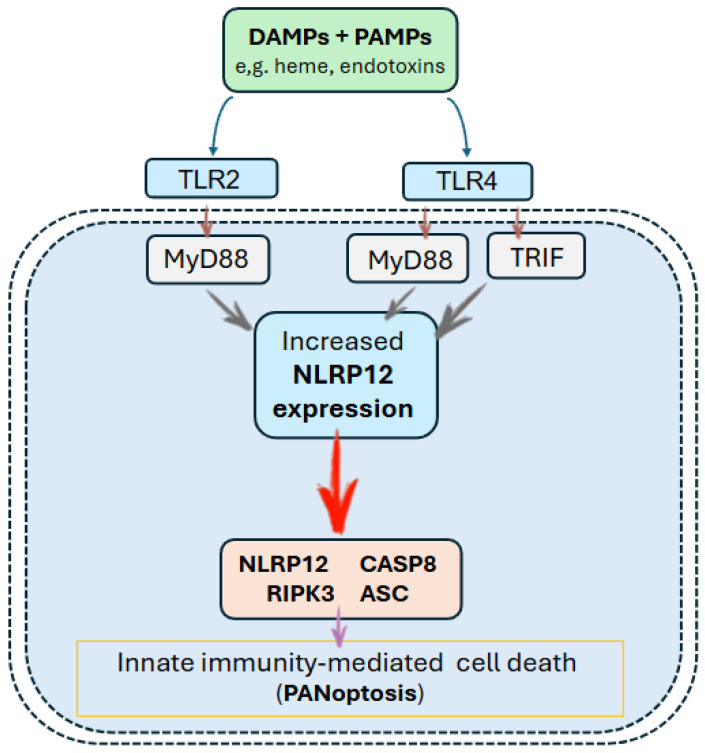
Representation of NLRP12-mediated PANoptosis. In response to cellular stress (either DAMPs or PAMPs), TLR2 and TLR4 signaling, primarily via MyD88 (with TRIF as the alternative TLR4 adaptor), induce NLRP12 expression, which then scaffolds ASC to recruit caspase-8 and RIPK3, thereby assembling the NLRP12-PANoptosome, which coordinates PANoptotic cell death.

Furthermore, there is evidence that NLRP12 can act as an immune sensor detecting a combination of damage-associated molecular patterns (or DAMPs, e.g., heme) and pathogen-associated molecular patterns (or PAMPs, e.g., endotoxins), driving assembly of the PANoptosome, a multiprotein complex containing ASC, RIPK3, caspase-8, and NLRP3, whose role is to trigger a lytic form of inflammatory cell death that integrates pathways of pyroptosis, apoptosis, and necroptosis (called PANoptosis) [17] (Figure 2).

With respect to the mutational spectrum of *NLRP12*, the first variants reported in 2008—p.Arg284* and an insertion causing a splicing defect—were loss-of-function variants, resulting in reduced NLRP12-mediated NF-κB inhibition [8]. Furthermore, in 2011, Jéru et al. showed in cell-based assays that NLRP12 could also be affected by gain-of-function variants, such as the p.Arg352Cys missense change, which increases NLRP3-inflammasome activity by promoting ASC speck formation and caspase-1 processing [10,18,19]. Different classes of variants may therefore differentially affect gene function and its crosstalk with other inflammatory mediators through distinct mechanisms, reflecting the overall complexity of innate immunity machinery.

Herein, we aim to discuss the molecular functions of NLRP12 and their relevance to SAIDs’ pathogenesis, focusing on genetic findings and subsequent mechanisms involved. Additionally, we aim to describe the clinical presentation of a cohort of 20 patients who were referred to our outpatient clinic of recurrent fevers (during the period of 2020–2025) and discriminate the spectrum of autoinflammatory signs and symptoms in reference to the genetic variants discovered.

## 2. Methods

### 2.1. Study Cohort and Patient Selection

This study included a cohort of 20 patients presenting with recurrent fevers and carrying *NLRP12* variants. All patients were evaluated at our periodic fever center at the Polyclinic between 2020 and 2025. Disease onset occurred during childhood or adolescence in most patients. Pediatric-onset cases were further assessed for PFAPA syndrome based on recurrent fever associated with lymphadenopathy, pharyngitis, and aphthous stomatitis.

Clinical manifestations were systematically recorded, including fever characteristics (peak temperature, duration, frequency), musculoskeletal symptoms (arthralgia, myalgia), gastrointestinal symptoms (abdominal pain, diarrhea), cutaneous manifestations (urticaria-like rashes, morbilliform eruptions, vasculitis-like lesions, livedo reticularis), adenitis/lymphadenopathy, aphthous stomatitis, pharyngitis/pharyngodynia, asthenia, headache, and hearing loss. Trigger factors such as cold exposure were documented when available. Comorbidities and prior medical history were also collected. Inflammatory markers, including erythrocyte sedimentation rate (ESR), C-reactive protein (CRP), and serum amyloid A (SAA), were assessed during febrile episodes when available, as well as outside flare periods for selected patients. Information on administered therapies was collected, including corticosteroids, NSAIDs, paracetamol, colchicine, anti–IL-1 biologics (anakinra, canakinumab), hydroxychloroquine, azathioprine, antihistamines, and probiotics.

### 2.2. Genetic Testing

All patients underwent *NLRP12* variant analysis using dedicated targeted next-generation sequencing (NGS) panels, as part of the routine diagnostic workup. A minimum sequencing coverage of 20× was required. Regions not reaching this threshold were validated by Sanger sequencing. Sequencing data were analyzed to identify single-nucleotide variants and small insertions or deletions in the *NLRP12* gene.

### 2.3. Study Design

This observational cohort study aimed to correlate clinical phenotypes with *NLRP12* variants, integrating clinical, laboratory, and treatment data to characterize the spectrum of autoinflammatory manifestations.

## 3. Clinical Features of Patients with NLRP12 Variants

The complete cohort of 20 patients, with main clinical features and *NLRP12* variants, is presented in Table 1.

Disease onset occurred during childhood or adolescence (<18 years) in 15 out of 20 patients (75% of the cohort), while all remaining patients developed symptoms between 18 and 40 years. Notably, five pediatric-onset cases (P3, P4, P8, P9, P11) presented with recurrent fever associated with lymphadenopathy, pharyngitis, and aphthous stomatitis and were therefore clinically classified as having periodic fever, aphthous stomatitis, pharyngitis, adenitis (PFAPA) syndrome. Recurrent fever was the most frequent clinical manifestation in the whole cohort, being reported in all but one patient. Eleven out of 20 patients experienced febrile episodes, with peak temperatures ranging from 38 to 41 °C. Both duration and frequency of febrile flares were highly variable. Most patients reported episodes lasting approximately 1–10 days; however, in a few cases, fever persisted for up to one month. Similarly, recurrence rates varied widely, ranging from 1–4 episodes per month to infrequent attacks occurring less than once per year. The latter pattern was observed in patient P14, who also experienced prolonged inflammatory episodes with fever.

Cold exposure as a trigger for inflammatory flares, one of the hallmark features of FCAS2, was reported in only 4 out of 20 cases (20%). However, this observation should be interpreted cautiously: many patients were children, and symptom reporting may be less reliable than for adults, as trigger-specific questions are not always addressed during history data collection. Fourteen out of 20 patients (70%) reported musculoskeletal manifestations, most commonly arthralgia and/or myalgia. Abdominal pain was present in 12 patients (60%), being associated with diarrhea in eight cases. Three patients also reported dysmenorrhea, with inflammatory symptoms worsening around the menstrual cycle, as already reported and known for FMF [20]. Cutaneous manifestations were observed in 10 cases (50%), with a broad spectrum of presentations: most patients had urticaria-like rashes, whereas others showed a morbilliform skin eruption. In two cases (P6 and P14), skin involvement was more severe, featuring vasculitis-like lesions. In one patient (P5), livedo reticularis was additionally observed. Adenitis and/or lymphadenopathy were reported in 12 cases (60%), while aphthous stomatitis was reported in 7 (35%). Our cohort also exhibited a wide spectrum of additional clinical manifestations, including asthenia (explicitly reported in patients P1, P7, P8 and P13), diarrhea (in patients 1, 4, 6, 7, 12, 13, 19 and 20) and headache (in patients 5, 6, 13, 15 and 18). Pharyngitis and pharyngodynia were observed in all five pediatric patients with PFAPA symptoms and in two further individuals (P7 and P20). Sensorineural hearing loss was documented only in P1. Patient P11, who also expressed HLA-B27 and HLA-B35, presented a history of ulcerative proctitis and perianal abscess, whereas P12 had poor weight gain, possibly related to the frequent diarrhea episodes. Notably, P12 reported onset of symptoms shortly after a vaccination.

Information on inflammatory markers was available for only 16 out of the 20 patients. Of these, 14 showed elevated erythrocyte sedimentation rate (ESR) and C-reactive protein (CRP) levels during “attacks”, whereas in patients P6, P15 and P19, an increase in serum amyloid-A (SAA) was documented. Data on inflammatory parameters outside flare periods were available for only eight patients (P3, P4, P5, P11, P12, P13, P17 and P19), in whom these markers were within normal ranges.

Regarding comorbidities, P1 had a concurrent diagnosis of coeliac disease and Hashimoto’s thyroiditis; P6 had acne, polycystic ovary syndrome (PCOS), and hypercholesterolemia, as well as Alternaria and olive allergy; P7 had selective IgA deficiency and history of biliary and renal calculi; P11 presented with polyarthritis; lastly, P18 and P20 had a concurrent diagnosis of fibromyalgia.

Regarding treatments administered to our patients, they mostly received anti-inflammatory agents: in more detail, ten patients were treated with corticosteroids, eight with non-steroidal anti-inflammatory drugs (NSAIDs) or paracetamol, five with colchicine, and six with anti–IL-1 biologic therapies (including both anakinra and canakinumab). In a single case, hydroxychloroquine and azathioprine were used (P19); in another patient, only antihistamines were used (P6), while in P12, the only recorded treatment was a probiotic.

## 4. Genetic Findings Regarding *NLRP12* Variants

All patients underwent genetic counseling and were tested using a next-generation sequencing (NGS) panel targeting genes associated with SAIDs. In most cases, sequencing was performed at our center by massively parallel sequencing on an Ion Torrent platform. The genes included in the panel were: *ALPK1*, *ADA2*, *IL1RN*, *LPIN2*, *MEFV*, *MVK*, *NLRC4*, *NLRP3*, *NLRP12*, *NOD2*, *PSMB8*, *PSTPIP1*, *TNFRSF1A*, and *TNFAIP3*. It should be noted that, for some patients, a more limited gene set was analyzed, because testing had been performed several years earlier, prior to subsequent updates of the panel. The same applies for individuals who were tested at other centers. Moreover, some patients in this cohort underwent whole-exome sequencing (however, the results of this analysis are currently not available).

All patients included in this study were found to carry one *NLRP12* variant; the characteristics of the variants are reported in Table 2.

Notably, all *NLRP12* variants detected, except one (c.289+6G>C in P6), were missense. Three variants were common between patients: c.1054C>T in P1 and P11, c.116G>T in P4, P5, P9 and P10, and c.1206C>G in P7, P8 and P11. For each variant, we assessed its population allele frequency using the gnomAD reference database (https://gnomad.broadinstitute.org/, accessed on 10 February 2026), and, as reported in Table 2, most are rare or very rare. Contrariwise, two variants, c.116G>T and c.1206C>G, that were shared by patients presented MAF > 5%. If available, their clinical classifications, as reported in the ClinVar database (https://www.ncbi.nlm.nih.gov/clinvar/, accessed on 10 February 2026), are included. Of the 16 variants identified, 2 were not reported in the ClinVar database (last accessed 10 February 2026): c.289+6G>C (P6) and c.673A>G (P3). The others were listed in ClinVar at the time of consultation as conflicting interpretation (CIP), variant of uncertain significance (VUS), benign (B) or likely benign (LB). For missense variants, the REVEL (Rare Exome Variant Ensemble Learner) prediction tool was used. REVEL integrates several prediction tools, including SIFT, PolyPhen-2, Mutation Assessor, PROVEAN, etc. It returns a score from 0 to 1 (the higher the value is, the greater the probability that the variant pathogenic is); in practice, >0.5 indicates a likely pathogenic (or probably damaging) variant. For the only intronic variant, c.289+6G>C (P6), the Splice AI tool was employed. It is a deep learning-based tool developed to predict the impact of genetic variants on RNA splicing and provides a score ranging from 0 to 1, where higher values indicate a greater probability that the variant affects splicing. Finally, 10 patients presented additional variants in other genes involved in autoinflammation, particularly within the *MEFV* gene, associated with FMF. These additional variants were both VUS and pathogenic, such as Met694Val. Their presence might have influenced the clinical phenotype compared to patients who carry only *NLRP12* variants.

**Figure 3:**
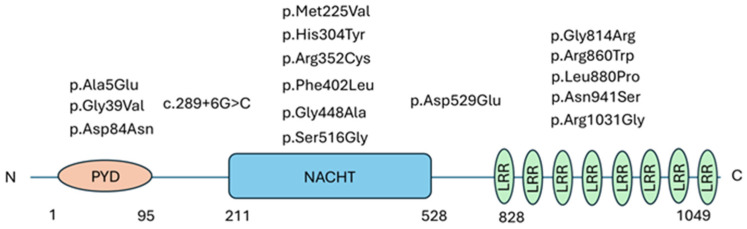
*NLRP12* functional domains and distribution of variants identified in our patient cohort. The figure illustrates the key structural sites of *NLRP12*, including the pyrin domain (PYD), NACHT domain, and leucine-rich repeat (LRR) regions, with patient-specific variants being mapped onto the corresponding domains. This scheme highlights potential correlations between variant location and clinical manifestations within our cohort of patients with *NLRP12* variants.

We mapped the identified variants onto the NLRP12 protein architecture. Domain boundaries were defined according to UniProt using the canonical isoform (UniProt P59046) (Figure 3).

Consistent with previous reports [4,5], most *NLRP12* variants were localized within the NACHT domain, a highly conserved functional region that is required for ATP-dependent oligomerization and activation of NLR proteins [10]. Specifically, we identified 15 missense variants: three mapped to the PYD, six to the NACHT domain, and five to the LRR region. In addition, we detected an intronic variant (c.289+6G>C) located at the +6 position of the 5′ splice-donor site, upstream of the NACHT domain. Lastly, we identified the c.1587C>G variant, resulting in p.(Asp529Glu) and mapping immediately downstream of the NACHT domain of *NLRP12* (UniProt P59046), in the inter-domain region between NACHT and the C-terminal LRRs. Interestingly, several variants recurred within our cohort: p.(Gly39Val) was identified in 4/20 patients, p.(Phe402Leu) in 3/20, and p.(Arg352Cys) in 2/20.

Lastly, to our knowledge, four patients (P11, P15, P18 and P19) inherited *NLRP12* variants from a first-degree relative. In particular, patient P11 inherited c.1054C>T from her unaffected mother and c.1206C>G from her unaffected father; in this family a maternal uncle of P11 presented recurrent fever without genetic testing analysis. Patient P15 inherited c.2822A>G (and also c.1772T>C in the *MEFV* gene) from her unaffected mother and c.2440G>A from her father who, up to the age of 16, had recurrent fevers and psoriasis. Patient P18 inherited the c.910C>T variant from her unaffected mother, and the c.1546A>G was inherited by patient P19 from her father, who had a history of recurrent fevers. Unfortunately, because of the retrospective design of the study, comprehensive data on the presence of *NLRP12* variants in first-degree relatives was not fully available. In addition, a positive family history of SAIDs was documented in patients P7 (mother with recurrent fever and renal insufficiency) and P13 (mother with recurrent pharyngotonsillitis), although no information on genetic testing was available.

## 5. Discussion

In this study, we describe a cohort of 20 patients recruited from the outpatient clinic of recurrent fevers within our Periodic Fevers Research Center at our university. All patients are carriers of *NLRP12* variants, identified through a targeted NGS panel dedicated to recurrent fevers. They presented a broad spectrum of autoinflammatory manifestations, with periodically recurring fever (19/20), musculoskeletal involvement (14/20), and gastrointestinal symptoms (12/20) as the most frequently represented. Notably, gastrointestinal manifestations, including abdominal pain and diarrhea, have been reported in a significant proportion of published cases related to *NLRP12* variants [5], plausibly given the role of NLRP12 in the intestinal immune homeostasis. Indeed, in murine models, NLRP12 acts as a negative regulator of inflammatory signaling, suppressing colon inflammation and tumorigenesis, through the inhibition of noncanonical NF-κB signaling [21]. Furthermore, there is evidence that NLRP12 prevents colon inflammation by maintaining gut microbiome and fostering growth of protective commensal taxa [22,23]. These data suggest that the gastrointestinal symptoms observed in *NLRP12* variant carriers in our cohort may reflect a potential alteration of mucosal inflammatory signaling involving the gut microbiota.

Another interesting finding is the recurrence of PFAPA-like symptoms in the cohort. PFAPA syndrome is observed in children (usually < 5 years), showing almost punctually recurring fevers every 4–6 weeks combined with at least one among aphthous stomatitis, pharyngitis and/or adenitis with cervical lymph node enlargement; patients remain asymptomatic between febrile flares, with both normal growth and overall development [24]. Even if both environmental factors and immunologic predisposition conspire to the pathogenesis of PFAPA, a possible genetic propensity should be considered. Furthermore, longitudinal follow-up of pediatric patients with PFAPA-like symptoms, extended to the transition to adulthood, may help clarify whether PFAPA in some cases could represent an early manifestation of a more complex autoinflammatory phenotype. The clinical course of P11, who carried two *in trans* *NLRP12* variants, is illustrative: after an initial diagnosis of PFAPA at 8 years, the patient subsequently developed a severe autoinflammatory picture characterized by cold-triggered flares with fever, arthromyalgia, polyarthritis, abdominal pain, adenitis, and cutaneous manifestations. In patient P15, the presence of two *in trans NLRP12* variants were associated with a non-typical clinical manifestation. In this landscape, the possible causative role of *NLRP12* variants should be further investigated.

Overall, two main clinical presentations may be delineated in our cohort: one that is more typical of *NLRP12* variants (i.e., cold-triggered flares and abdominal pain) and the other that is less typical (i.e., PFAPA-like features), in which an oligogenic pathogenesis may be envisioned. This scenario outlined an autoinflammatory condition with a broad spectrum of clinical findings, in which recurrent fever represents the main symptom. Notably, four patients inherited *NLRP12* variants from a first-degree relative. These observations provide some insight into familial transmission, but the presence of unaffected carriers and the VUS classification of many variants highlight the complexity of genotype–phenotype correlations and the challenges of interpreting family segregation. Furthermore, a clear correlation between variant type and clinical phenotype does not appear to emerge. For instance, among patients with a PFAPA phenotype, *NLRP12* variants include both very common variants that are generally classified as benign or likely benign and rarer variants that are classified as CIP, suggesting that the presence or type of variant alone may not be sufficient to explain the observed clinical variability. A clear genotype–phenotype correlation in this cohort of patients was not observed. Thus, our findings support the hypothesis that the mechanism by which *NLRP12* variants work should not be limited to its strictly monogenic role in autoinflammation, rather than to a possible contributing factor. As shown by previous scientific reports [4], the majority of *NLRP12* variants detected were missense and not equally distributed along the gene sequence, with a higher number of variants mapping within the NACHT domain. However, the spectrum of variants that we detected is different from the *NLRP12* variants originally associated with NLRP12-AID/FCAS2, which were mostly deleterious changes, including nonsense and splice-site variants [8,18]. We also identified an intronic splice-region variant (c.289+6G>C) located at the +6 position of the 5′ donor splice site, whose impact on splicing efficiency or creation of a cryptic splice site remains unclear. Targeted functional assays are necessary to solve uncertainties about the possible pathogenicity of this variant, hopefully in combination with exome analysis. Furthermore, compared with the review reported by Vatandoost et al. [5], our cohort includes several additional variants that are not previously described in that series, with only five variants overlapping with those reported by [5], i.e., c.1054C>T, c.1206C>T, c.910C>T and c.1343G>C. Overall, our results further expand the mutational spectrum associated with *NLRP12*.

Recently, Yun et al. introduced the concept of genetically transitional disease (GTD) to describe an intermediate inheritance model bridging monogenic and polygenic diseases, in which a genetic variant may be necessary but not sufficient to determine a full clinical expression [25]. This innovative “disease model” supports a new interpretation of VUS in SAIDs, positioning this case in a “gray zone” between clearly pathogenic and fully benign findings [25]. This applies to several *NLRP12* variants identified in our cohort: for example, the recurrent allele p.Phe402Leu, which is present in three out of 20 cases of our cohort, has been previously reported both in subjects with autoinflammatory manifestations and in healthy controls, supporting a model of low penetrance and/or modifier effect rather than a fully penetrant classic monogenic disease [26,27]. The detection of *NLRP12* variants in asymptomatic first-degree relatives provides additional support for this hypothesis, suggesting that *NLRP12* variants may act as a predisposing genetic background for autoinflammation, with clinical features effectively manifesting only in the presence of additional genetic risk-factors and/or environmental triggers [4]. Moreover, nine of the 20 patients carried additional variants in other genes that are implicated in inflammatory pathways, and their potential contribution to phenotype modulation warrants further investigation.

The most recent clinical and genetic research in the field of FMF has demonstrated the role of gain-of-function inflammation, even in the presence of heterozygous variants in the *MEFV* gene, especially when the variants involve critical domains, such as exon 10. Similarly, clinical cases demonstrating some variants in the *NLRP12* gene that have not yet been classified show an activation of inflammatory pathways and are compatible with the hypothesis of the role of gain-of-function, as already demonstrated for FMF patients [28].

Furthermore, the occurrence of comorbidities such as coeliac disease, Hashimoto’s thyroiditis and ulcerative proctitis with perianal abscesses, polyarthritis and fibromyalgia is consistent with the role of NLRP12, in the presence of HLA predisposing genes, to potentially amplify immune pathways, leading to autoimmune comorbidities. In fact, previous anecdotal reports [1,4] have highlighted coexisting autoimmune features and immune deficiencies among *NLRP12* variant carriers, suggesting NLRP12 dysfunction as a player supporting both autoinflammation and susceptibility to autoimmunity disorders [8,29].

Finally, concerning the therapeutic approach, we observed marked heterogeneity in the use of anti-inflammatory medications. Colchicine produced partial efficacy in P15 (2+), (who also carried MEFV:c.1772T>C); thus, the therapeutic plan now included canakinumab. Furthermore, colchicine showed no positive effect in P11 and caused a worsening of diarrhea in P7; finally, P20 had a good response to the combination of colchicine and anakinra. Only six out of 20 patients received anti–IL-1 biologic drugs, despite the evidence that IL-1 blockade may represent a particularly effective therapeutic target [30]. In this context, a more in-depth characterization of the contribution of *NLRP12* variants to the autoinflammatory phenotype could represent a clinically meaningful decision-support tool, facilitating the choice of more targeted and personalized therapeutic strategies.

## 6. Limits of the Study

The number of patients (20) included in this cohort may seem low, but it becomes significant considering that this disease is largely considered ultrarare, with a frequency of <1 case per 1,000,000. However, these data require multicenter collaboration studies to gather further clinical observations and correlate them with the different genetic mutations reported. Given the complexities associated with SAIDs in patients with *NLRP12* variants, it will be necessary to deepen our understanding of the role of regulatory pathways underpinning innate immunity processes through interdisciplinary partnerships.

## 7. Conclusions and Future Research

A cohort of 20 patients recruited from the Periodic Fevers Research Center of our university were shown to be carriers of *NLRP12* variants, which were identified through a targeted NGS panel dedicated to investigating recurrent fevers. The patients presented a broad spectrum of autoinflammatory manifestations, with periodically recurring fever (19/20), musculoskeletal involvement (14/20), and gastrointestinal symptoms (12/20). Notably, gastrointestinal manifestations (abdominal pain and diarrhea) have been reported in a significant proportion of cases, given the role of NLRP12 in intestinal immune homeostasis. Indeed, there is evidence that NLRP12 prevents colon inflammation, and our data suggest that the gastrointestinal symptoms observed in *NLRP12* variant carriers may reflect a potential alteration of inflammatory signaling involving the gut microbiota. Furthermore, the occurrence of autoimmune comorbidities is consistent with the role of NLRP12 as a player in priming both autoinflammation and susceptibility to autoimmunity disorders.

## Figures and Tables

**Table 1:** List of main clinical findings described in the cohort of 20 patients with *NLRP12* variants.

ID	Age of Onset	Fever	N° of Episodes (Duration)	Cold Trigger	Arthromyalgia	Abdominal Pain	Cutaneous Manifestations	Adenitis	Aphthous Stomatitis	Therapy
P1	25 y	+	3/year	+	+	+	nr	nr	+	corticosteroids, NSAIDs
P2	<18 y	+	2/month (3–4 days)	nr	+	+	+	+	nr	canakinumab
P3	6 y	+	1/month (3–6 days)	nr	nr	nr	nr	+	+	corticosteroids
P4	<18 y	+	1/month (3–6 days)	nr	nr	nr	nr	+	+	corticosteroids
P5	<1 y	+	nr	nr	+	+	nr	nr	nr	NSAIDs
P6	13 y	−	(1–3 weeks)	nr	+	+	++	nr	nr	antihistamines
P7	40 y	+	1/month (3–4 days)	+	+	+	nr	+	+	colchicine, anakinra, tonsillectomy
P8	<18 y	+	2/month (2–5 days)	−	+	−	−	+	nr	corticosteroids, NSAIDs
P9	2 y	+	1/month	nr	nr	−	−	+	−	corticosteroids, colchicine, tonsillectomy
P10	2 y	+	1–4/month	nr	+	+	nr	nr	nr	corticosteroids, NSAIDs, colchicine
P11	8 y	+	2/month	+	+	+	+	+	−	anakinra, colchicine, tonsillectomy
P12	9 m	+	nr (1–10 days)	−	nr	+	nr	+	−	probiotics
P13	18 y	+	3–4/year (1–2 days)	nr	−	+	−	−	−	corticosteroids
P14	18 y	+	<1/year (>1 month)	nr	nr	nr	+	nr	nr	corticosteroids, NSAIDs
P15	6 y	+	1–3/month (24 h)	nr	+	nr	+	nr	nr	colchicine, paracethamol, canakinumab
P16	15 y	+	1/month (4–15 days)	nr	+	nr	+	+	nr	nr
P17	4 y	+	(10–30 days)	nr	+	+	+	+	+	corticosteroids, NSAIDs
P18	15 y	+	1/month	+	+	+	+	+	+	NSAIDs
P19	18 y	+	1/month (10 days)	nr	+	+	+	nr	nr	corticosteroids, NSAIDs, hydroxychloroquine, azathioprine, canakinumab
P20	16 y	+	1/month (7–21 days)	nr	+	nr	+	+	+	colchicine, anakinra

nr = not reported; age at onset: m = months; y = years; PCOS = polycystic ovary syndrome; NSAIDs = nonsteroidal anti-inflammatory drugs.

**Table 2:** List of the *NLRP12* variants in our cohort of 20 patients. For each patient, the variant with its consequent protein change is reported, with allele frequency being estimated by GnomAD, ClinVar classification, effects being predicted using REVEL or SpliceAI tools (ver. 1.3.1), and the presence of variants in other genes involved in autoinflammation being assessed.

ID	*NLRP12* Variant	Protein Change	Allele Frequency	ClinVar Classification	Prediction	Other Variants Found
P1	c.1054C>T	p.Arg352Cys	0.000287	CIP (3 VUS, 5 LB)	REVEL: 0.545	nr
P2	c.1587C>G	p.Asp529Glu	0.00002664	VUS	REVEL: 0.210	nr
P3	c.673A>G	p.Met225Val	0.000003098	nr	REVEL: 0.376	nr
P4	c.116G>T	p.Gly39Val	0.2175	B/LB	REVEL: 0.231	nr
P5	c.116G>T	p.Gly39Val	0.2175	B/LB	REVEL: 0.231	NLRP3:c.2107C>A, p.(Gln703Lys)
P6	c.289+6G>C	p.?	0.00001177	nr	SpliceAI: 0.14	ADAR:c.577C>G, p.(Pro193Ala)
P7	c.1206C>G	p.Phe402Leu	0.06492	B/LB	REVEL: 0.373	MEFV:c.442G>C, p.(Glu148Gln)
P8	c.1206C>G	p.Phe402Leu	0.06492	B/LB	REVEL: 0.373	MEFV:c.605G>A, p.(Arg202Gln) MEFV:c.605G>A, p.(Arg202Gln) NOD2:c.721C>T, p.(Pro241Ser) MVK: c.769-38C>T in homozygous
P9	c.116G>T	p.Gly39Val	0.2175	B/LB	REVEL: 0.231	PSMB8:c.22G>A,p.(Gly8Arg) MEFV:c.605G>A, p.(Arg202Gln)
P10	c.116G>T	p.Gly39Val	0.2175	B/LB	REVEL: 0.231	PSTPIP1:c.484C>T, (p.Ala162Val) in heterozygous MEFV:c.2080A>G, p.(Met694Val) MEFV:c.605G>A, p.(Arg202Gln) NOD2:c.2782G>A, p.(Val928Ile)
P11	c.1054C>T; c.1206C>G	p.Arg352Cys; p.Phe402Leu	0.000287; 0.06492	CIP; B/LB	REVEL: 0.545; REVEL: 0.373	nr
P12	c.2639T>C	p.Leu880Pro	0.000009916	VUS	REVEL: 0.590	nr
P13	c.2578C>T	p.Arg860Trp	0.00009542	CIP (5 VUS, 1 B)	REVEL: 0.127	MEFV:c.605G>A, p.(Arg202Gln)
P14	c.3091C>G	p.Arg1031Gly	0.0004659	CIP (2 VUS, 1 LB, 3 B)	REVEL: 0.119	nr
P15	c.2440G>A; c.2822A>G *	p.Gly814Arg; p.Asn941Ser	0.00002293; 0.00002788	VUS; VUS	REVEL: 0.0530; 0.141	MEFV:c.1772T>C, p.(Ile591Thr)
P16	c.14C>A	p.Ala5Glu	0.0001716	CIP (1 VUS, 1 B, 3 LB)	REVEL: 0.0760	nr
P17	c.250G>A	p.Asp84Asn	0.000006196	VUS	REVEL: 0.356	nr
P18	c.910C>T	p.His304Tyr	0.003837	CIP (1 VUS, 5 B, 4 LB)	REVEL: 0.740	ADA2:c.2T>C, p.(Met1?)
P19	c.1546A>G	p.Ser516Gly	0.00001053	VUS	REVEL: 0.619	nr
P20	c.1343G>C	p.Gly448Ala	0.00007623	CIP (5 VUS, 1 LB)	REVEL: 0.240	MEFV:c.2084A>G,p.(Lys695Arg) NLRC4:c.2629G>A, p.(Val877Met)

CIP = conflicting interpretation of pathogenicity; VUS = variant of uncertain significance; LB = likely benign; B = benign; nr = not reported. * Referred to NM_00001277126.2.

## Data Availability

No new data were created or analyzed in this study.
